# Comparing the psychometric properties of the EQ-5D-3L and EQ-5D-5L descriptive systems and utilities in atopic dermatitis

**DOI:** 10.1007/s10198-022-01460-y

**Published:** 2022-04-12

**Authors:** Kamilla Koszorú, Krisztina Hajdu, Valentin Brodszky, Alex Bató, L. Hunor Gergely, Anikó Kovács, Zsuzsanna Beretzky, Miklós Sárdy, Andrea Szegedi, Fanni Rencz

**Affiliations:** 1 Department of Dermatology, Venereology and Dermatooncology, Semmelweis University, Budapest, Hungary; 2 Károly Rácz Doctoral School of Clinical Medicine, Semmelweis University, Budapest, Hungary; 3 Department of Dermatological Allergology, University of Debrecen, Debrecen, Hungary; 4 Department of Dermatology, Faculty of Medicine, University of Debrecen, Debrecen, Hungary; 5 Department of Health Economics, Corvinus University of Budapest, 8 Fővám tér, Budapest, 1093 Hungary

**Keywords:** EQ-5D-3L, EQ-5D-5L, Atopic dermatitis, Psychometrics, Utility, I10

## Abstract

**Background:**

Atopic dermatitis (AD) is a common chronic inflammatory skin disorder affecting up to 10% of adults. The EQ-5D is the most commonly used generic preference-accompanied measure to generate quality-adjusted life years (QALYs) for economic evaluations.

**Objectives:**

We aimed to compare psychometric properties of the three-level and five-level EQ-5D (EQ-5D-3L and EQ-5D-5L) in adult patients with AD.

**Methods:**

In a multicentre cross-sectional study, 218 AD patients with a broad range of severity completed the EQ-5D-3L, EQ-5D-5L, Dermatology Life Quality Index (DLQI) and Skindex-16. Disease severity outcomes included the Investigator Global Assessment, Eczema Area and Severity Index and the objective SCORing Atopic Dermatitis.

**Results:**

A good agreement was established between the two EQ-5D versions with an intraclass correlation coefficient of 0.815 (95% CI 0.758–0.859, *p* < 0.001). Overall, 33 different health state profiles occurred in the EQ-5D-3L and 84 in the EQ-5D-5L. Compared to the EQ-5D-3L, ceiling effect was reduced for the mobility, self-care, usual activities and pain/discomfort dimensions by 4.6–11.5%. EQ-5D-5L showed higher average relative informativity (Shannon’s evenness index: 0.64 vs. 0.59). EQ-5D-5L demonstrated better convergent validity with EQ VAS, DLQI and Skindex-16. The two measures were similar in distinguishing between groups of patients based on disease severity and skin-specific quality of life with a moderate or large effect size (*η*^2^ = 0.083–0.489).

**Conclusion:**

Both instruments exhibited good psychometric properties in AD; however, the EQ-5D-5L was superior in terms of ceiling effects, informativity and convergent validity. We recommend the use of the EQ-5D-5L to measure health outcomes in clinical settings and for QALY calculations in AD.

## Introduction

Atopic dermatitis (AD) is a common chronic inflammatory skin disorder affecting up to 10% of adults [1, 2]. It can appear on any area of the body, but predilection sites are the face, hands, and flexural surfaces of the extremities [1]. Clinical symptoms include recurrent eczematous lesions and intense itch that may considerably decrease patients’ health-related quality of life (HRQoL). The excessive dryness, itching and scratching may cause substantial limitations in daily functioning, social interactions, leisure activities, and may lead to sleep disturbance [3–6]. Treatments include topical emollients, topical corticosteroids, calcineurin inhibitors and systemic immunosuppressants (e.g. corticosteroids, cyclosporine A, azathioprine, mycophenolate mofetil or methotrexate), according to disease severity [7, 8]. Recently, an increasing number of new treatment options have become available for moderate-to-severe AD, such as targeted biological therapies (dupilumab and tralokinumab) and small molecules (baricitinib, abrocitinib and upadacitinib) [9].

AD represents a large burden on patients and society with an average annual total cost per patient of up to €20,000 [10–12]. New treatments typically require more health resources, and thus, providing evidence on their cost-effectiveness is important to show their value for money and to support financial decision-making in healthcare [13]. In these economic evaluations, quality-adjusted life year (QALY) is used as a summary outcome that combines quantity and quality of life. Using generic preference-accompanied instruments is the most common way to assess HRQoL to generate QALYs. These measures consist of a descriptive system and a set of utility values [14]. The most commonly used generic preference-accompanied measure is the EQ-5D [15]. Over the past three decades, it has been used in over 10,000 studies and by now, it has become a preferred instrument to estimate QALYs in pharmacoeconomic guidelines in nearly 30 countries [16–18].

The EQ-5D has two versions for adults, the three-level (EQ-5D-3L, hereafter 3*L*) [19], and the five-level (EQ-5D-5L, hereafter 5*L*) [20]. Both have been increasingly used in dermatological patient populations [21–24]. The major difference between the two adult EQ-5D questionnaires is that the 5*L* includes not three, but five levels in each dimension and uses a standardised wording across dimensions. In many countries, including Hungary, both adult questionnaires are recommended by pharmacoeconomic guidelines [18]; however, these may lead to different cost-effectiveness outcomes, therefore understanding their psychometric properties in different contexts and settings is critical to inform the debate about the choice of instrument.

Several previous studies in different health condition groups and general population samples showed improved measurement properties of the 5*L* descriptive system, such as reduced ceiling effect, better informativity and construct validity [25, 26]. Among dermatological conditions, the measurement properties of the 3*L* and 5*L* have been compared in psoriasis [27] and hidradenitis suppurativa (HS) [28]; however, no comparative study is available in AD. There could be large differences in how the descriptive systems perform across different health conditions, even among chronic skin diseases. Furthermore, it is important to examine how measurement properties of the descriptive systems translate into the discriminatory power of utilities, as this has a direct impact on QALYs.

This study therefore seeks to compare the psychometric properties of the 3*L* and 5*L* with regard to both the descriptive systems and utilities (hereafter referred to as index scores in the context of the EQ-5D) in adult patients with AD. The Hungarian 3*L* and 5*L* value sets will be used to estimate index scores that were developed in a parallel valuation study using the same respondents (*n* = 1000), protocol (i.e. EQ-VT), valuation method (i.e. composite time trade-off) and modelling approach (i.e. heteroscedastic Tobit) [29]. This will give us a unique opportunity to compare not only the descriptive systems but also utilities using real-world patient data. We aim to focus on the following psychometric properties: ceiling and floor effect, agreement, redistribution properties, informativity, convergent and known-groups validity.

## Methods

### Study design and patients

Between March 2018 and January 2021, a cross-sectional, multicentre study was conducted in Hungary among consecutive adult AD patients. Data were collected at two university dermatology clinics in Budapest and Debrecen and an outpatient centre in Pannonhalma. In each study site, patients were asked to read and sign an informed consent form on paper before participating in the study. Ethical approval was granted by the Scientific and Ethical Committee of the Medical Research Council in Hungary (reference No.: 29655/2018/EKU). Eligible patients were aged 18 years or over and had a diagnosis of AD confirmed by a dermatologist. Patients completed multiple generic and skin-specific HRQoL measures in a fixed order: Dermatology Life Quality Index (DLQI) [30], EQ-5D-5L (5L) [20], Skindex-16 [31], and EQ-5D-3L (3L) [19]. The 5*L* was placed before the 3*L* within the questionnaire to prevent the underuse of the second and fourth levels in the 5*L* [32]. The EuroQol visual analogue scale (EQ VAS) was completed only once, as part of the 5*L*.

### Measures

A detailed description of the HRQoL measures used in the study is provided in Table 1, including their items, response levels, scoring and interpretation. In addition to HRQoL instruments, patients were asked to assess their level of itching and sleep disturbance for the past 1 month and their current disease severity (PtGA) using 11-point visual analogue scales (VAS). Demographic and medical history data were obtained from patients, including age, sex, education, employment, family history of AD and disease duration. Dermatologists assessed patients’ disease severity using the Investigator Global Assessment (IGA) [33], the objective SCORing Atopic Dermatitis (oSCORAD) [34], and the Eczema Area and Severity Index (EASI) scales [35], and provided information about treatments. These severity scales are widely used in clinical trials, treatment guidelines and core outcome sets [36–38]. We used the cut-off values for the interpretation of EASI and oSCORAD scores as published in Chopra et al. [39], and for DLQI as suggested by Hongbo et al. [40, 41].

**Table 1:** Generic and skin-specific HRQoL measures used in the study

Type of measure	Measure	Structure, number of items	HRQoL areas covered	Response options	Scoring and interpretation	Recall period
Generic	EQ-5D-5L [20]	Descriptive system: 5 dimensions (5 items) EQ VAS^a^: a 20-cm vertical scale	Descriptive system: -Mobility -Self-care -Usual activities -Pain/discomfort -Anxiety/depression EQ VAS: -Perception of current health status	In each dimension of the descriptive system: -1: No problems -2: Slight problems -3: Moderate problems -4: Severe problems -5: Extreme problems/unable to EQ VAS: 0–100 scale -0: Worst health you can imagine -100: Best health you can imagine	-Descriptive system: 5^5^ = 3125 possible health state profiles (e.g. 11,111 = no problems in each dimension; 55,555 = most severe problems in each dimension) -A utility (− 0.848 to 1) is assigned to these profiles based on the Hungarian value set (1: full health; negative values: worse than dead) [29] EQ VAS 0–100 (Higher scores represent better HRQoL)	Today
Generic	EQ-5D-3L [19]	Descriptive system: 5 dimensions (5 items)	Descriptive system: -Mobility -Self-care -Usual activities -Pain/discomfort -Anxiety/depression	In each dimension of the descriptive system: -1: No problems -2: Some/moderate problems -3: Extreme problems/unable to/confined to bed	-Descriptive system: 3^5^ = 243 possible different health state profiles (e.g. 33,333 = most severe problem in each dimension) -A utility (− 0.865 to 1) is assigned to these profiles based on the Hungarian value set (1: full health; negative values: worse than dead) [29]	Today
Skin-specific	Dermatology Life Quality Index (DLQI) [30]	10 items	-Symptoms -Feelings -Daily activities -Leisure -Work/school -Personal relationships -Treatment	0: Not at all *or* not relevant 1: A little 2: A lot 3: Very much	Item scores are added up to yield a DLQI total score that ranges from 0 to 30 (Higher scores represent worse HRQoL) Interpretation according to Hongbo et al. [40]: - 0–1: no effect on patient's life - 2–5: small effect on patient's life - 6–10: moderate effect on patient's life - 11–20: very large effect on patient's life - 21–30: extremely large effect on patient's life	Last 7 days
Skin-specific	Skindex-16 [31]	3 subscales, 16 items altogether	3 subscales: -Symptoms (4 items) -Emotions (7 items) -Functioning (5 items)	A 7-point bipolar rating scale for each item -0: Never bothered -6: Always bothered	-Subscale scores: scores of the corresponding items are summed up and transformed to a scale of 0–100 -Skindex-16 total score: averaging the scores of the three subscales (Higher scores represent worse HRQoL)	Last 7 days

*EQ VAS* EuroQol visual analogue scale, *HRQoL* health-related quality of life

^a^The EQ VAS of the EQ-5D-3L was not used in the present study

### Statistical analyses

We built on methods established in previous psychometric studies comparing the performance of the 3*L* and 5*L* across different healthy and patient populations [25, 27, 28, 32, 42].

#### Feasibility and ceiling

The feasibility was assessed by comparing the number of missing responses for the two EQ-5D questionnaires. Missing values were not imputed. Due to the two additional response levels, a reduced ceiling was expected in the 5*L* compared to the 3*L*. First, we computed the difference in the proportion of respondents scoring no problems (absolute ceiling reduction). Then, we calculated the relative reduction as (ceiling_3L_-ceiling_5L_)/ceiling_3L_. We compared the difference in ceiling between the 3*L* and 5*L* using McNemar’s test. The distributions of index scores were visualised using histograms, and the proportion of patients reporting no problems across all five EQ-5D dimensions was calculated to estimate the ceiling.

#### Agreement

The difference between 3*L* and 5*L* index scores was tested by Wilcoxon signed-rank test. The agreement between the 3*L* and 5*L* was displayed using a Bland–Altman plot [43], with the mean of the 3*L* and 5*L* index scores on the axis *x* and their difference on axis *y*. The 95% confidence interval for the difference was calculated as the mean difference ± 1.96 × standard deviation (SD). The points outside the upper and lower limit were considered outliers. We used the intraclass correlation coefficient (ICC) to test parallel forms reliability, which reflects both the agreement and degree of correlation between the two descriptive systems [44]. A two-way random model with absolute agreement was used to estimate ICCs [45]. We classified ICC values as follows: poor: 0–0.39, fair: 0.40–0.59, good: 0.60–0.74 and excellent: 0.75–1 [46]. Good or excellent agreement was expected between 3*L* and 5*L* [25].

#### Redistribution properties

We calculated the proportion of consistent and inconsistent 3*L* -5*L* response pairs using cross-tabulations. A 5*L* response at least two levels away from its 3*L* pair was considered inconsistent (e.g. respondent chooses severe problems [level 4] on the 5*L* and some problems [level 2] on the 3*L*) [32]. To calculate the average size of inconsistency, 3*L* responses were recoded to a 5*L* scale (level 1_3L_ = level 1_5L_, level 2_3L_ = level 3_5L_ and level 3_3L_ = level 5_5L_) and the following formula was used: |3*L*-5*L*| – 1 [32].

#### Informativity

Informativity reflects the ability of an instrument to discriminate between different levels of health [47]. The informativity of the five dimensions of 3*L* and 5*L* was determined using Shannon’s (*H*′) and Shannon’s evenness (*J*′) indices [47, 48]. The *H*′ expresses the absolute information content (the number of possible responses) combined with how evenly the information is distributed across all responses, while *J*′ represents the evenness of distribution exclusively. Our hypothesis was that the 5*L* with its two additional levels improves the informativity of the 3*L* [49]. We calculated the two indices according to the following formulae (*L*: number of levels in one dimension of the EQ-5D; p_i_: percentage of patients choosing the *i*th level):

$${H}^{\prime}=- \sum_{i=l}^{L}{p}_{i}{\mathrm{log}}_{2}{p}_{i}$$

$$J^{\prime } = \frac{{H^{\prime } }}{{H^{\prime } _{{{\text{max}}}} }},{\text{where }}H_{{\max }}^{\prime } = {\text{log}}_{{\text{2}}} L$$

Higher *H*′ indicates better informativity (range: 0 to log_2_*L*, where log_2_*L* is 1.85 for the 3*L* and 2.32 for the 5*L*). The value of *J*′ ranges from 0 to 1, whereby 0 corresponds to the worst discriminatory power, when all responses are in the same response level and 1 indicates the best discriminatory power with even distribution of responses among all levels [25].

#### Convergent validity

Convergent validity was analysed by calculating Spearman’s rank order correlation coefficients (*r*_s_) between the 3*L* and 5*L* dimensions and index scores and previously validated other measures. Based on earlier studies, we hypothesized at least moderate correlations between the EQ-5D dimensions and index scores and EQ VAS, DLQI, and Skindex-16 [50], and weak correlations with severity measures, including IGA, oSCORAD, EASI, and PtGA VAS [51]. In general, we expected most EQ-5D dimensions and index scores to correlate weakly or very weakly with sleep disturbance and itching VAS, as these are not parts of the EQ-5D descriptive system [52]. The only exception was itching for which we assumed a moderate correlation with the pain/discomfort dimension [53]. We expected the 5*L* to be more strongly related to these disease severity and skin-specific HRQoL measures. We interpreted correlation coefficients as follows: very weak < 0.20, weak 0.20–0.39, moderate 0.40–0.59, strong 0.60–0.79 and very strong 0.80 < [54].

#### Known-group validity

Due to the skewed distribution of EQ-5D index scores, non-parametric Mann–Whitney and Kruskal–Wallis tests were used to assess and compare the ability of 3*L* and 5*L* to distinguish between known groups of patients defined by severity scores on IGA, oSCORAD, and EASI or skin-specific HRQoL on DLQI. We hypothesized that patients with higher disease severity or worse skin-specific HRQoL have significantly lower index scores and the 5*L* is able to better differentiate across known groups.

Effect sizes (ES) were calculated as follows:

$$\mathrm{ES}\left(Z\right)=\frac{\mathrm{Mann}\text{-Whitney} \, {\text{Z}}}{n-1}$$

$$\mathrm{ES}\left(H\right)=\frac{\mathrm{Kruskal}\text{-Wallis} \, {\text{H}} \, -k+1}{n-k}$$

where *k* is the number of groups, and *n* is the sample size. We interpreted ESs ≥ 0.01 as small, ≥ 0.06 as moderate and ≥ 0.14 as large [55]. Relative efficiency (RE) was computed as the ratio of the ESs of 5*L* and 3*L* index scores. A RE larger than 1 indicated that the 5*L* was more efficient in distinguishing between known groups. Data analysis was carried out in R Statistical Software (v4.1.2 Vienna, Austria) [56]. A *p* < 0.05 was considered statistically significant and all tests were two-sided.

## Results

Overall, 224 adult AD patients were invited to the study, four of whom declined to participate and another two patients did not finish the questionnaire. Thus, a total of 218 patients completed the questionnaire. No respondents were excluded from the data analysis. Demographic and clinical characteristics of patients are summarized in Table 2. Overall, 57.8% were women and the mean age was 31.3 ± 11.7 years (range 18–73). According to oSCORAD, 21.1%, 33.5% and 45.4% had clear/mild, moderate and severe AD, respectively. Nearly two-thirds of the patients (63.3%) were treated by systemic non-biological therapy at the time of the survey, while 23.4% received topical therapy only and a minority (9.6%) were untreated. Patients reported substantial impairment in their skin-specific HRQoL with mean DLQI score of 13.4 ± 8.5 and Skindex-16 total score of 56.8 ± 27.5 (Table 3).

**Table 2:** Demographic and clinical characteristics of patients with AD

Variables	Mean (SD) or *n* (%)
Age (years)	31.34 (11.68)
Sex	
Female	126 (57.8%)
Male	92 (42.2%)
Education (missing = 2)	
Primary	12 (5.6%)
Secondary	112 (51.9%)
Tertiary	92 (42.6%)
Employment^a^	
Employed full-time	109 (50.0%)
Employed part time	24 (11.0%)
Retired	7 (3.2%)
Disability pensioner	6 (2.8%)
Unemployed	12 (5.5%)
Student	60 (27.5%)
Other	23 (10.6%)
Disease duration (years) (missing = 3)	19.02 (12.91)
Current treatment	
None	21 (9.6%)
Topical (only)	51 (23.4%)
Phototherapy	3 (1.4%)
Systemic non-biological^b^	138 (63.3%)
Biological (dupilumab)	5 (2.3%)

*AD* atopic dermatitis

^a^More than one response could be marked

^b^Including antimicrobial treatment. Monotherapy or in combination with topical or phototherapy

**Table 3:** Disease severity and health-related quality of life scores of AD patients

Variables	Mean (SD)	Median (Q1-Q3)
EQ-5D-3L index (− 0.865 to 1)	0.85 (0.15)	0.85 (0.80–1.00)
EQ-5D-5L index (− 0.848 to 1)	0.82 (0.22)	0.89 (0.78–0.97)
EQ VAS (0–100) (missing = 1)	69.15 (20.50)	75.00 (57.00–85.00)
DLQI (0–30)	13.44 (8.46)	14.00 (6.00–20.00)
Skindex-16 total (0–100)	56.84 (27.46)	61.49 (35.64–80.04)
Symptoms subscale (0–100)	62.44 (29.64)	68.75 (37.50–87.50)
Emotions subscale (0–100)	61.21 (29.18)	69.05 (40.48–85.71)
Functioning subscale (0–100)	46.87 (31.48)	46.67 (20.00–74.17)
Itching VAS (0–10) (past one month) (missing = 1)	7.01 (2.92)	8.00 (5.00–9.00)
Sleeping VAS (0–10) (past one month) (missing = 3)	5.51 (3.53)	6.00 (2.00–9.00)
PtGA VAS (0–10) (missing = 1)	6.04 (2.74)	7.00 (4.00–8.00)
oSCORAD (0–83)	35.91 (14.61)	36.90 (26.60–46.73)
EASI (0–72)	15.76 (11.99)	14.40 (6.10–21.98)
IGA (0–5)	2.77 (1.04)	3.00 (2.00–3.00)

For EQ-5D-3L, EQ-5D-5L and EQ VAS higher scores refer to better health status. For all other measures higher scores represent worse health status

*DLQI* Dermatology Life Quality Index, *EASI* Eczema Area and Severity Index, *IGA * Investigator Global Assessment, *oSCORAD* objective Scoring Atopic Dermatitis, *PtGA VAS* Patient’s Global Assessment of disease severity visual analogue scale

### Feasibility and ceiling

There were no missing responses across the 3*L* or 5*L* descriptive systems; however, one patient left the EQ VAS blank. A total of 33 different health states were reported on the 3*L* and 84 on the 5*L*.

The frequencies and percentages of patients reporting a ceiling are presented in Table 4. A statistically significant reduction in ceiling effect between 5*L* and 3*L* was observed in the mobility (4.6%), self-care (11.5%) and usual activities (9.2%) dimensions, while in the anxiety/depression dimension the ceiling slightly increased (2.3%), although the difference between 3*L* and 5*L* was insignificant. The largest relative ceiling effect reduction was found for usual activities (17.2%), followed by self-care (13.2%) and pain/discomfort (12.8%). The proportion of patients reporting no problems in each dimension (11111) demonstrated a reduction from 27.5% on the 3*L* to 22.5% on the 5*L* (*p* = 0.029). There were a total of 6 (2.8%) ‘best health you can imagine’ (= 100) responses on the EQ VAS.

**Table 4:** Ceiling effect, inconsistencies and informativity of the EQ-5D-3L and EQ-5D-5L in AD

Dimensions	Ceiling effects							Inconsistencies		Informativity			
	EQ-5D-3L		EQ-5D-5L		Ceiling effect reduction		McNemar’s test *p*-value			EQ-5D-3L		EQ-5D-5L	
	*n*	Ceiling (*n*, %)	*n*	Ceiling (*n*, %)	Absolute (%)	Relative (%)		Inconsistent response pairs (*n*, %)^a^	Average size of inconsistencies	*H*′	*J*′	*H*′	*J*′
Mobility	218	192 (88.1%)	218	182 (83.5%)	4.59	5.21	0.016	3 (1.4%)	1.00	0.53	0.33	0.81	0.35
Self-care	218	190 (87.2%)	218	165 (75.7%)	11.47	13.16	< 0.001	9 (4.1%)	1.11	0.55	0.35	1.07	0.46
Usual activities	218	116 (53.2%)	218	96 (44.0%)	9.17	17.24	0.002	15 (6.9%)	1.07	1.15	0.73	1.83	0.79
Pain/discomfort	218	86 (39.4%)	218	75 (34.4%)	5.05	12.79	0.054	17 (7.8%)	1.12	1.17	0.74	1.98	0.85
Anxiety/depression	218	99 (45.4%)	218	104 (47.7%)	− 2.29	− 5.05	0.404	20 (9.2%)	1.15	1.27	0.80	1.70	0.73
Overall (11111) or average	–	60 (27.5%)	–	49 (22.5%)	5.05	18.33	0.029	64 (5.9%)	1.09	0.93	0.59	1.48	0.64

*H′* Shannon’s index, *J′* Shannon’s evenness index

^a^The total number of pairs is 218 for all dimensions

### Agreement

**Fig. 1:**
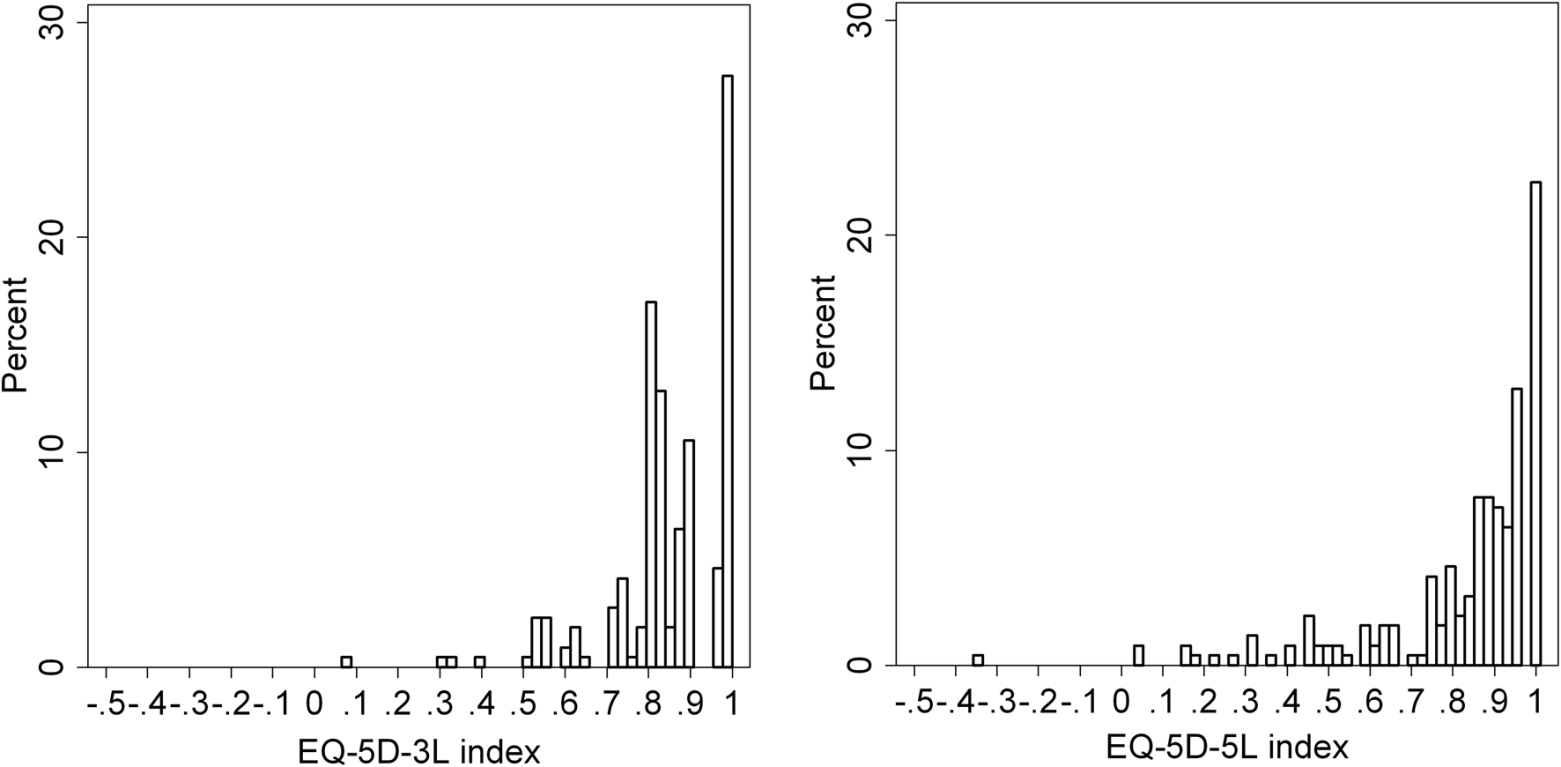
Distribution of EQ-5D-3L and EQ-5D-5L index scores in AD patients. *AD* atopic dermatitis

**Fig. 2:**
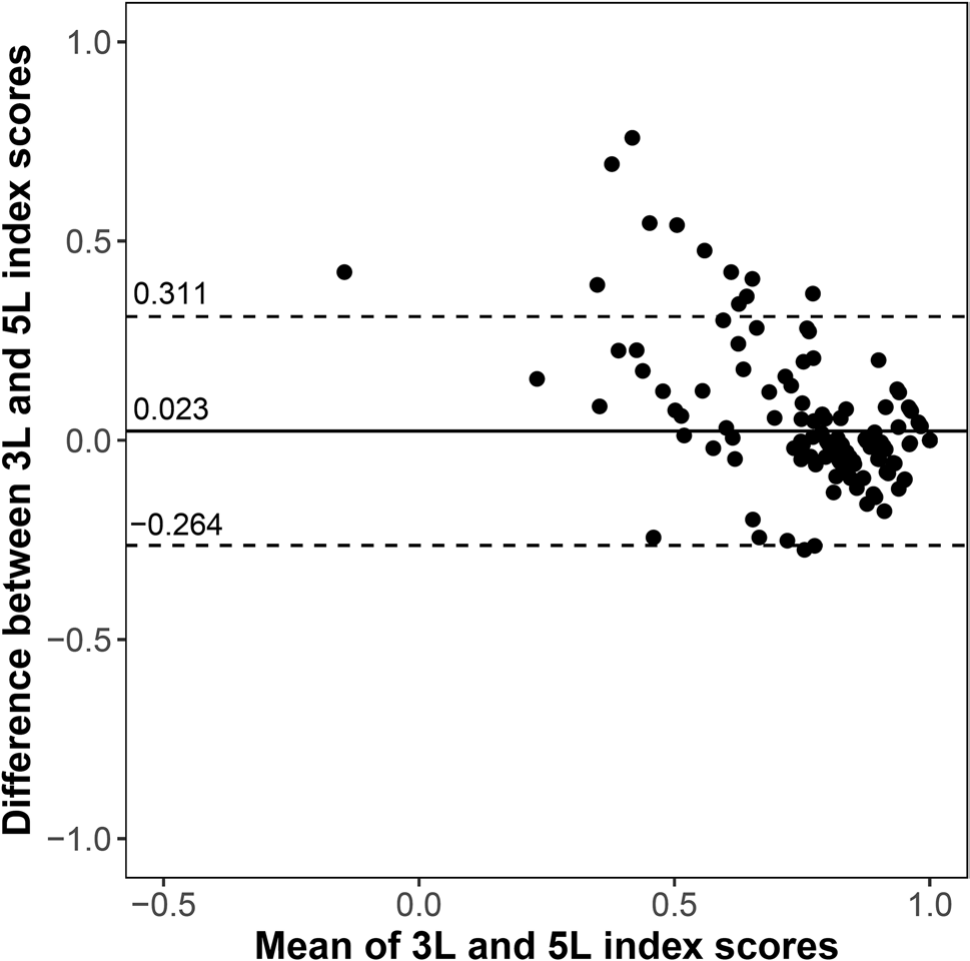
Bland–Altman plot of EQ-5D-3L and EQ-5D-5L index scores in AD patients. *AD* atopic dermatitis

The distribution of 3*L* and 5*L* index scores is shown in Fig. 1. One patient had a negative index score on the 5*L*, while no negative values were observed on the 3*L*. The mean 5*L* index score was lower than that of the 3*L*, although the difference was insignificant (0.82 ± 0.22 vs. 0.85 ± 0.15, *p* = 0.928). An overall good agreement was observed between the two measures with an ICC of 0.815 (95% CI 0.758–0.859, *p* < 0.001). This was confirmed by the Bland–Altman plot in Fig. 2. The differences between 3*L* and 5*L* index scores tended to be higher for more severe health states.

### Redistribution properties and inconsistencies

The percentages of consistent and inconsistent response pairs in each level of 3*L* and 5*L* are shown in Table 5. A total of 64 (5.9%) inconsistent response pairs were reported by 50 (22.9%) patients. The average size of inconsistency was very small (1.09). The highest proportion of inconsistent response pairs (9.2%) and largest average inconsistency (1.15) were present in the anxiety/depression dimension (Table 4). The fewest inconsistent responses occurred in the mobility dimension (1.4%) with an average size of 1.00.

**Table 5:**
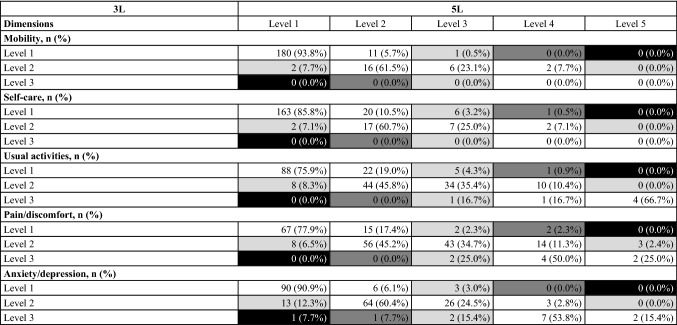
Redistribution properties: cross-tabulation of EQ-5D-3L and EQ-5D-5L responses

The size of inconsistency is represented in grayscale with more inconsistency in darker fields. White fields contain consistent response pairs. Percentages may not total 100 by row due to rounding

### Informativity

The informativity results are provided in Table 4. The 5*L* increased the absolute (*H*′) informativity across all dimensions (3*L* 0.53–1.27 vs. 5*L* 0.81–1.98) suggesting the usefulness of the two additional response levels in the 5*L*. Relative informativity (*J*′) increased for the first four dimensions (3*L* 0.33–0.74 vs. 5*L* 0.35–0.85), but not for the anxiety/depression (3*L* 0.80 vs. 5*L* 0.73).

### Convergent validity

Table 6 shows the correlations between EQ-5D dimensions and index scores with other instruments and scales. The results provide support for most of our hypotheses. The EQ-5D mobility and self-care dimensions showed weak or no correlations with other measures. The usual activities, pain/discomfort and anxiety/depression dimensions were moderately or strongly correlated with the DLQI and Skindex-16 subscale and total scores (*r*_s_ = 0.429–0.670). The only exception was the symptoms subscale of Skindex-16 that correlated weakly with anxiety/depression. As expected, the itching experienced in the past 1 month exhibited the strongest correlation with the pain/discomfort dimension (3*L* 0.351 vs. 5*L* 0.476). Similarly, sleep VAS score for the past 1 month correlated moderately with pain/discomfort (3*L* 0.381 vs. 5*L* 0.484). Both itching and sleep VAS showed weak correlations with 3*L* and moderate correlations with 5*L* index scores.

**Table 6:** Convergent validity: Spearman’s correlation coefficients

Outcome measures	EQ-5D						
	Version	Mobility	Self-care	Usual activities	Pain/discomfort	Anxiety/depression	Index score
EQ VAS (0–100)	3L	− 0.255	− 0.246	− 0.483	− 0.547	− 0.507	0.626
	5L	− 0.333	− 0.316	− 0.495	− 0.676	− 0.531	0.665
DLQI (0–30)	3L	0.267	0.338	0.570	0.557	0.509	− 0.669
	5L	0.354	0.376	0.651	0.670	0.545	− 0.731
Skindex-16 total (0–100)	3L	0.181	0.237	0.526	0.515	0.521	− 0.622
	5L	0.302	0.329	0.566	0.657	0.556	− 0.684
Skindex-16 symptoms (0–100)	3L	0.136	0.136	0.429	0.488	0.380	− 0.513
	5L	0.217	0.212	0.459	0.612	0.392	− 0.572
Skindex-16 emotions (0–100)	3L	0.105*	0.195	0.429	0.444	0.511	− 0.549
	5L	0.248	0.260	0.460	0.551	0.531	− 0.574
Skindex-16 functioning (0–100)	3L	0.239	0.298	0.535	0.492	0.510	− 0.619
	5L	0.339	0.375	0.594	0.617	0.556	− 0.691
Itching VAS (0–10) (past one month)	3L	0.096*	0.106*	0.299	0.351	0.305	− 0.383
	5L	0.186	0.159	0.379	0.476	0.361	− 0.452
Sleeping VAS (0–10) (past one month)	3L	0.147	0.091*	0.312	0.381	0.276	− 0.397
	5L	0.167	0.195	0.364	0.484	0.368	− 0.479
IGA (0–5)	3L	0.171	0.130*	0.241	0.326	0.236	− 0.328
	5L	0.207	0.202	0.289	0.389	*0.223*	− 0.349
EASI (0–72)	3L	0.098*	0.113*	0.171	0.254	0.244	− 0.274
	5L	0.196	0.128*	0.215	0.328	0.262	− 0.308
oSCORAD score (0–83)	3L	0.174	0.139	0.236	0.312	0.271	− 0.342
	5L	0.273	0.226	0.280	0.375	*0.254*	− 0.359
PtGA VAS (0–10)	3L	0.176	0.215	0.437	0.487	0.385	− 0.531
	5L	0.286	0.301	0.503	0.586	0.405	− 0.583

Italic values indicate a lower correlation coefficient for the 5*L* compared to the 3*L*

*DLQI* Dermatology Life Quality Index, *EASI* Eczema Area and Severity Index, *IGA * Investigator Global Assessment *oSCORAD* objective Scoring Atopic Dermatitis, *PtGA VAS* Patient’s Global Assessment of disease severity visual analogue scale

*p  ≥ 0.05

Moderate or strong correlations were detected between the EQ-5D index scores and DLQI and Skindex-16 total scores (*r*_s_ = − 0.731 to − 0.622). Both the 3*L* and 5*L* index scores produced strong correlations with the EQ VAS (*r*_s_ =  0.626 vs. 0.665). Contrary to our hypotheses, weak correlations were observed between index scores and disease severity measured by IGA, oSCORAD, and EASI (*r*_s_ = − 0.359 to − 0.274). PtGA VAS scores showed moderate correlations with 3*L* and 5*L* index scores (*r*_s_ = − 0.531 vs. − 0.583). With very few exceptions, the 5*L* demonstrated stronger correlations with all instruments and scales. The difference between the 3*L* and 5*L* was particularly pronounced for the pain/discomfort dimension.

### Known-group validity

Results on known-group validity analyses are presented in Table 7. Both the 3*L* and 5*L* were able to distinguish across predefined groups of patients based on severity and skin-specific HRQoL (i.e. DLQI score bands) with moderate to large effect sizes (0.080–0.489). Patients with more severe disease and worse skin-specific HRQoL had lower EQ-5D index scores (*p* < 0.001). The 5*L* more efficiently discriminated across EASI (RE 1.033) and DLQI groups (RE 1.275), while the 3*L* slightly outperformed 5*L* in the case of IGA (RE 0.978) and oSCORAD groups (RE = 0.966).

**Table 7:** Known-groups validity of EQ-5D-3L and EQ-5D-5L index scores in AD patients

Outcome measures	EQ-5D-5L					EQ-5D-3L					RE^b^
	*n*	Mean (SD)	Median (Q1-Q3)	*p*-value^a^	ES	*n*	Mean (SD)	Median (Q1–Q3)	*p*-value^a^	ES	
IGA											
Clear	5	0.95 (0.09)	1.00 (0.96–1.00)	< 0.001	0.105	5	0.97 (0.07)	1.00 (1.00–1.00)	< 0.001	0.108	0.978
Minimal	27	0.93 (0.11)	0.97 (0.91–1.00)			27	0.93 (0.11)	1.00 (0.88–1.00)			
Mild	32	0.91 (0.08)	0.92 (0.87–0.96)			32	0.87 (0.09)	0.88 (0.80–0.96)			
Moderate	108	0.82 (0.20)	0.88 (0.76–0.96)			108	0.85 (0.13)	0.82 (0.80–0.96)			
Marked	41	0.70 (0.32)	0.83 (0.49–0.92)			41	0.78 (0.20)	0.80 (0.72–0.90)			
Severe	5	0.77 (0.07)	0.79 (0.75–0.80)			5	0.70 (0.15)	0.72 (0.55–0.82)			
EASI											
Clear (0.0–0.1)	5	0.80 (0.33)	0.96 (0.80–1.00)	< 0.001	0.083	5	0.93 (0.11)	1.00 (0.85–1.00)	< 0.001	0.080	1.033
Mild (0.2–5.9)	46	0.92 (0.10)	0.96 (0.88–1.00)			46	0.91 (0.10)	0.96 (0.82–1.00)			
Moderate (6–22.9)	118	0.82 (0.21)	0.90 (0.77–0.96)			118	0.85 (0.14)	0.88 (0.80–0.96)			
Severe (23–72)	49	0.74 (0.28)	0.85 (0.67–0.92)			49	0.78 (0.18)	0.80 (0.78–0.85)			
oSCORAD											
Clear (0–7.9)	8	0.97 (0.07)	1.00 (0.99–1.00)	< 0.001	0.099	8	0.98 (0.05)	1.00 (1.00–1.00)	< 0.001	0.103	0.966
Mild (8–23.9)	38	0.91 (0.11)	0.94 (0.87–1.00)			38	0.90 (0.10)	0.93 (0.82–1.00)			
Moderate (24–37.9)	73	0.86 (0.17)	0.92 (0.81–0.97)			73	0.86 (0.12)	0.88 (0.80–0.96)			
Severe (38–83)	99	0.76 (0.26)	0.85 (0.65–0.92)			99	0.80 (0.17)	0.82 (0.78–0.90)			
DLQI											
No effect (0–1)	15	0.99 (0.01)	1.00 (1.00–1.00)	< 0.001	0.489	15	0.99 (0.03)	1.00 (1.00–1.00)	< 0.001	0.384	1.275
Small effect (2–5)	37	0.96 (0.05)	1.00 (0.93–1.00)			37	0.94 (0.09)	1.00 (0.90–1.00)			
Moderate effect (6–10)	40	0.93 (0.08)	0.94 (0.92–0.96)			40	0.91 (0.07)	0.90 (0.88–1.00)			
Very large effect (11–20)	76	0.80 (0.17)	0.84 (0.76–0.92)			76	0.82 (0.11)	0.82 (0.80–0.88)			
Extremely large effect (21–30)	50	0.62 (0.29)	0.66 (0.45–0.85)			50	0.73 (0.19)	0.80 (0.62–0.82)			

*DLQI* Dermatology Life Quality Index, *EASI* Eczema Area and Severity Index, *ES* effect size, *IGA * Investigator Global Assessment, *oSCORAD* objective Scoring Atopic Dermatitis, *RE* relative efficiency;

^a^Mann–Whitney test or Kruskal–Wallis test, where a *p* < 0.05 was considered statistically significant

^b^Relative efficiency compared to the EQ-5D-3L

## Discussion

This is the first study to compare the psychometric properties of the two adult versions of the EQ-5D in patients with AD. Both the 3*L* and 5*L* exhibited overall good psychometric properties in AD; however, the 5*L* was superior in terms of ceiling effects, informativity and convergent validity. Previously, similar head-to-head 3*L* vs. 5*L* comparative studies were carried out in two other chronic inflammatory dermatological conditions, psoriasis and hidradentitis suppurativa also in Hungary that allow for direct comparisons. In line with these prior studies of similar sample size, the 5*L* resulted in a much richer set of responses with more than twice as many unique health state profiles (psoriasis 86 vs. 30 [27], hidradenitis suppurativa 101 vs. 43 [28], and AD 84 vs. 33). Further similarities across these studies include a substantial relative ceiling effect reduction with the 5*L* (psoriasis 11.4%, hidradenitis suppurativa 14.6% and AD 18.3%), the low proportion of inconsistent response pairs (psoriasis 3.9%, hidradenitis suppurativa 8.0% and AD 5.9%) and an identical or improved average relative informativity of the 5*L* (psoriasis 0.61, hidradenitis suppurativa 0.74 and AD 0.64). It seems therefore that the two extra levels of the 5*L* are effectively used in AD similarly to other chronic dermatological diseases and enable patients to more commonly report health problems.

The improved measurement properties of the 5*L* descriptive system appear to be translated to the level of utilities as 5*L* index scores showed stronger correlations with disease severity and skin-specific HRQoL measures in AD in comparison with the 3*L*. The exceptionally strong correlations of the 5*L* index scores with Skindex-16 (*r*_s_ = − 0.684) and DLQI (*r*_s_ = − 0.731) total scores lend supportive evidence to the excellent validity of the 5*L* in this patient population. However, validity between known disease severity and skin-specific HRQoL groups was established for both 3*L* and 5*L* with negligible difference in effect sizes between the two measures.

Several findings of the present study may be explained by the slightly different wording used in the 3*L* questionnaire compared to the 5*L*. Some of these changes affect all language versions (e.g. most severe level of mobility is ‘confined to bed’ in the 3*L* and ‘unable to walk’ in the 5*L*) and there are a few variations used solely in the Hungarian versions (e.g. the descriptor ‘anxiety/depression’ in the 5*L* is ‘anxiety/feeling down’ in the 3*L*) [29]. This latter modification seems to be responsible for the unexpected psychometric properties of the anxiety/depression dimension, including an increase in ceiling effect (3*L* 45.4% vs. 5*L* 47.7%), lower relative informativity of the 5*L* (3*L* 0.80 vs. 5*L* 0.73) and the highest rate of inconsistent response pairs in anxiety/depression (9.2%) among the five dimensions. Similar psychometric properties of the anxiety/depression dimension were reported by other studies from Hungary [27, 28, 57].

Itching is considered a hallmark symptom of AD that may adversely affect patients’ HRQoL, including sleep. It is currently debated to what extent the EQ-5D descriptive system is able to capture itching and sleep problems. A recent study with AD patients found very weak and insignificant correlation between 3*L* index scores and sleep disturbance as measured on weekly average scores of an 11-point numeric rating scale [58]. In another study with burn patients, the pain/discomfort dimension of the 5*L* showed moderate correlations with a 10-point itching VAS [53]. Recent qualitative evidence in psoriasis patients also suggests that the discomfort element of the pain/discomfort composite dimension may cover itching to a minor extent [59]. Our findings in AD showed a weak correlation for the 3*L* and a moderate for the 5*L* pain/discomfort dimension and index scores with itching and sleep problems. However, a 1-month recall period was used for the itching and sleep VAS, whereas the EQ-5D asks about ‘today’. These results are also relevant for the currently expanding bolt-on research programme for the EQ-5D. Over the past three decades, several additional dimensions (bolt-ons) have been developed for the EQ-5D to improve accuracy and precision of the measure in specific populations [60]. Among them, there is a bolt-on aiming to assess sleep problems and another psoriasis-specific bolt-on with two items, one of which, skin irritation measures the level of itching experienced by the respondent [61, 62].

Another noteworthy finding of this study is that mean index scores were lower in the 5*L* (5*L* 0.82 vs. 3*L* 0.85). As the Hungarian 3*L* and 5*L* value sets were developed in a parallel valuation study from a common sample, using the same preference elicitation method and modelling approach, the differences found in index scores reflect the wording differences between the two measures. The difference in mean 3*L* and 5*L* index scores was smaller at the top end of the scale near ‘full health’ and there was a widening gap at lower mean index scores. For example, patients with severe AD according to their oSCORAD score had considerably higher mean 3*L* index score than that in the 5*L* (0.80 vs. 0.76). In contrast, the difference was much smaller in either the ‘clear’ (0.98 vs. 0.97) or ‘mild’ (0.90 vs 0.91) oSCORAD groups. As a result, an assumed improvement from ‘severe’ to ‘clear’ skin may lead to a mean index score gain of 0.18 with the 3*L* and 0.21 with the 5*L* that might guarantee a lower cost-effectiveness ratio with the 5*L* for the same AD treatment.

The main strengths of the present study include the multicentre design, the diverse patient population in terms of sociodemographic and clinical background and the wide range of validated skin-specific HRQoL instruments and disease severity scales used. Potential limitations include the cross-sectional design that did not allow to assess test–retest reliability and responsiveness of the instruments. Furthermore, most patients were recruited at university clinics, where patients with mild disease may be underrepresented. Lastly, albeit the DLQI and Skindex-16 have been extensively validated in AD patients and are the most widely used HRQoL questionnaires in dermatological conditions [63, 64], these are skin-specific and not condition-specific instruments and their adequacy has been subject to criticism [65–69]. Further research may concentrate on the validation of the EQ-5D against AD-specific HRQoL measures, such as the Quality of Life Index for Atopic Dermatitis (QoLIAD) [70].

In summary, both the 3*L* and 5*L* showed an overall good validity in adult AD patients. The superiority of the 5*L* was confirmed in many aspects, including ceiling effect, informativity and convergent validity. Given the high prevalence and considerable societal burden of AD, our findings fill in an important gap in evidence needed when selecting instruments for economic evaluations. Such analyses have become particularly important with the increasing number of costly new therapies for AD, including biological and small molecule treatments [71–73]. Based on our findings, we recommend the use of the 5*L* to measure health outcomes both in clinical settings and for QALY calculations in adult AD.
